# Association between HALP score and in-hospital mortality in sepsis patients: a multicenter retrospective cohort study with external validation

**DOI:** 10.3389/fpubh.2025.1710118

**Published:** 2026-01-12

**Authors:** Xingping Lv, Xiaobin Liu, Yezhou Shen, Chen Li, Tuo Shen, Yusong Wang, Qimin Ma, Wei Zhou, Shaolin Ma, Feng Zhu

**Affiliations:** Department of Critical Care Medicine, Shanghai East Hospital, School of Medicine, Tongji University, Shanghai, China

**Keywords:** HALP score, in-hospital mortality, prognosis, risk stratification, sepsis

## Abstract

**Background:**

The HALP (Hemoglobin, Albumin, Lymphocyte, and Platelet) score integrates key parameters reflecting nutritional and immune status. However, its prognostic value for in-hospital mortality in sepsis patients remains underexplored.

**Objective:**

To investigate the association between HALP score and in-hospital mortality in sepsis patients using two large critical care databases.

**Methods:**

We conducted a retrospective cohort study including adult patients with Sepsis-3 from the eICU Collaborative Research Database (derivation cohort, *n* = 12,899) and the MIMIC-IV database (validation cohort, *n* = 3,726). HALP was calculated as (hemoglobin × albumin × lymphocyte count)/platelet count, using first available values upon ICU admission. Restricted cubic spline (RCS) models assessed nonlinear relationships between HALP and mortality. Kaplan–Meier survival curves and multivariable Cox regression models, adjusted for demographics, comorbidities, laboratory values, and Acute Physiology Score III, evaluated survival differences between low- and high-HALP groups. Segmented Cox regression examined associations below and above RCS-derived thresholds.

**Results:**

A total of 16,625 patients were analyzed. RCS analysis demonstrated significant nonlinear associations between HALP and in-hospital mortality in both cohorts (overall *P* < 0.001; nonlinearity *P* < 0.001 in eICU, *P* = 0.002 in MIMIC-IV), with an inflection point near 12.45. Below this threshold, each unit increase in HALP was associated with a 3% reduction in mortality risk (eICU: HR 0.97, 95% CI 0.95–0.99, *P* = 0.002; MIMIC-IV: HR 0.97, 95% CI 0.94–0.99, *P* = 0.008). Kaplan–Meier analyses showed significantly higher survival in the high HALP group (eICU: log-rank *P* = 0.005, HR 0.882, 95% CI 0.808–0.962; MIMIC-IV: log-rank *P* < 0.001, HR 0.723, 95% CI 0.607–0.862). Multivariable Cox regression confirmed that high HALP remained independently protective after full adjustment (eICU: HR 0.90, 95% CI 0.82–0.98, *P* = 0.017; MIMIC-IV: HR 0.85, 95% CI 0.74–0.98, *P* = 0.028).

**Conclusion:**

The HALP score demonstrates robust prognostic value for predicting in-hospital mortality in sepsis patients, with consistent nonlinear relationships validated across two large databases. Its simplicity and reliance on routine laboratory parameters support potential clinical application in sepsis risk stratification.

## 1 Introduction

Sepsis, a life-threatening condition characterized by dysregulated immune response to infection, remains a leading cause of mortality in intensive care units worldwide, with in-hospital mortality rates of 15–30% (1–3). The heterogeneous nature of sepsis pathophysiology and its rapid progression underscore the critical importance of early risk stratification to guide therapeutic interventions and optimize patient outcomes (4). Current prognostic tools, including APACHE II (5) and SOFA scores (6), demonstrate reasonable predictive performance but are limited by computational complexity and may not fully capture the multifaceted pathophysiological derangements characteristic of sepsis.

The hemoglobin, albumin, lymphocyte, and platelet (HALP) score, which integrates hemoglobin, albumin, lymphocyte count, and platelet count, offers a potentially valuable approach for assessing nutritional and immune status in critical illness (7). Initially developed as a prognostic marker in oncology, the HALP score has shown promise in various cancers, including gastric, urological, and esophageal malignancies (8–10). Recent studies have also explored its utility in other conditions, such as cardiovascular diseases (11, 12), suggesting its broader applicability as a prognostic tool. However, its prognostic value in sepsis, a condition characterized by systemic inflammation and immune dysregulation, remains underexplored.

This study evaluates the association between HALP score and in-hospital mortality among sepsis patients using the eICU Collaborative Research Database, with external validation in the MIMIC-IV database. We hypothesize that lower HALP scores are associated with increased mortality risk through a nonlinear dose-response relationship and aim to identify optimal cutoff values for clinical risk stratification. By leveraging two large critical care databases, this investigation will provide robust evidence regarding the prognostic value of the HALP score in sepsis management.

## 2 Methods

### 2.1 Study design and data sources

This retrospective cohort study utilized two large, well-established critical care databases to evaluate the prognostic value of the HALP score in sepsis patients. The primary analysis was conducted using the eICU Collaborative Research Database (eICU-CRD), which contains de-identified data from over 200,000 ICU admissions across 208 hospitals in the United States between 2014 and 2015 (13, 14). External validation was performed using the Medical Information Mart for Intensive Care IV (MIMIC-IV 2.2) database, which includes comprehensive clinical data from ICU admissions at Beth Israel Deaconess Medical Center in Boston, Massachusetts, spanning from 2008 to 2019 (15, 16). Both databases have been approved for research use and are freely accessible to qualified researchers who complete appropriate data use agreements.

### 2.2 Study population

#### 2.2.1 Inclusion criteria

Adult patients (≥18 years of age) with sepsis were identified using Sepsis-3 criteria (17), which was operationalized through a combination of ICD-9 and ICD-10 diagnostic codes indicating infection along with clinical evidence of organ dysfunction. Patients were required to have complete laboratory data for all four HALP score components (hemoglobin, albumin, lymphocyte count, and platelet count) available within 24 h of ICU admission to ensure accurate risk assessment during the critical early period of sepsis management. Patient identification and SOFA ≥ 2 calculation used structured PostgreSQL queries on the original databases (full reproducible SQL script in Supplementary material S1). Laboratory units were harmonized (hemoglobin and albumin in g/L; lymphocytes and platelets in × 10^9^/L).

#### 2.2.2 Exclusion criteria

Patients were excluded if they were younger than 18 years of age, had an ICU length of stay less than 24 h (to ensure adequate opportunity for clinical assessment), had missing data for any component of the HALP score calculation, or represented readmissions to the ICU (only the first ICU admission was included to avoid bias from repeated measurements). Identical inclusion and exclusion criteria were applied to both databases to ensure comparability of results between the derivation and validation cohorts.

### 2.3 HALP score calculation

The HALP score was calculated using the following formula (9):


$$\begin{array}{l} \text{HALP }=\;(\text{Hemoglobin }[\text{g}/\text{L}]\times \text{Albumin }[\text{g}/\text{L}]\times \\ \text{Lymphocyte count }[\times 10^{9}/\text{L}])/\text{Platelet count }[\times 10^{9}/\text{L}] \end{array}$$

Using the first available laboratory values upon ICU admission (typically within 0–6 h) to capture baseline severity without confounding by early interventions such as fluid resuscitation or antibiotics. Laboratory units were harmonized across databases: hemoglobin and albumin in g/L; lymphocytes and platelets in × 10^9^/L (full SQL code for extraction and conversion in Supplementary material S1). This approach aligns with established practices in critical care prognostication and ensures that the HALP score reflects the patient's initial condition during the acute phase of sepsis, minimizing treatment-related bias.

### 2.4 Outcome measures

The outcome was in-hospital mortality, defined as death occurring during the index hospitalization regardless of location within the hospital.

### 2.5 Covariates and confounding variables

To account for potential confounding factors, analyses were adjusted for clinically relevant variables including demographic characteristics (age and sex), laboratory parameters (white blood cell count, blood glucose, lactate, international normalized ratio, creatinine, and blood urea nitrogen), comorbidities (hypertension and diabetes mellitus), and illness severity as measured by the Acute Physiology Score III (APS III). These covariates were selected based on their established associations with sepsis outcomes and their availability in both databases.

### 2.6 Statistical analysis

Continuous variables were reported as median with interquartile range (IQR) and compared between groups using the Mann-Whitney U test due to non-normal distributions typical of clinical data. Categorical variables were presented as frequencies with percentages and compared using chi-square tests or Fisher's exact tests when appropriate.

Restricted cubic splines (RCS) analysis, based on Cox proportional hazards models, was performed to evaluate the nonlinear relationship between HALP score and mortality in both the eICU and MIMIC-IV databases. The RCS models were adjusted for the aforementioned covariates to account for potential confounding factors. Knots were placed at the 5th, 35th, 65th, and 95th percentiles of the HALP distribution following Harrell's recommended default settings for restricted cubic splines. Two types of nonlinear relationships were assessed: (1) the association between HALP score and hazard ratio, and (2) the association between HALP score and survival probability. Knots for the RCS models were selected based on optimal fit criteria to capture the nonlinearity of these associations effectively.

Optimal HALP cutoff values (inflection point) were determined from the RCS analysis to stratify patients into high and low HALP groups for each database. These cutoff values were identified at the inflection points of the spline curves where the relationship between HALP score and mortality risk demonstrated the most significant changes.

Subsequently, Kaplan-Meier (KM) survival analysis was conducted to compare survival probabilities between the high and low HALP groups in both the eICU and MIMIC-IV cohorts. Log-rank tests were used to assess statistical significance of survival differences between groups. Cox proportional hazards regression models were employed to evaluate the association between HALP score groups and mortality outcomes, with results reported as hazard ratios (HR) with 95% confidence intervals (CI). Multivariable Cox regression was performed using a multi-model strategy: Model 1 (crude/unadjusted), Model 2 (adjusted for demographics and comorbidities: age, gender, hypertension, diabetes mellitus), Model 3 (further adjusted for white blood cell count and glucose), and Model 4 (fully adjusted for all covariates: age, gender, hypertension, diabetes mellitus, white blood cell count, glucose, lactate, blood urea nitrogen, serum creatinine, international normalized ratio, prothrombin time, and APS III).

To further explore the relationship between HALP score and mortality, segmented Cox proportional hazards models were used to assess HALP as a continuous variable below and above the identified thresholds (12.4548 for eICU and 12.4551 for MIMIC-IV). In these models, in-hospital mortality was the outcome, hospital length of stay was the time variable, and adjustments were made for the same covariates (age, sex, white blood cell count, glucose, lactate, international normalized ratio, blood urea nitrogen, serum creatinine, hypertension, diabetes, and APS III). Results were reported as hazard ratios per unit increase in HALP score with corresponding 95% confidence intervals and *P*-values.

To evaluate potential collinearity between HALP and covariates, we calculated variance inflation factors (VIFs) and generated pairwise correlation matrices in both the eICU and MIMIC cohorts. Multicollinearity was considered absent if VIF < 5 and |r| < 0.3.

### 2.7 Statistical analysis

The proportional hazards assumption was evaluated using Schoenfeld residuals. Sensitivity analyses included (1) stratification by lactate tertiles, (2) exclusion of lactate from the model, and (3) competing-risk analysis using the Fine-Gray subdistribution hazard model treating live discharge as a competing event for in-hospital mortality.

All statistical analyses were performed using R software (version 4.5.1) with appropriate packages for survival analysis and spline modeling. The *P*-value of less than 0.05 was considered statistically significant for all analyses.

## 3 Results

### 3.1 Study population and baseline characteristics

**Figure 1:**
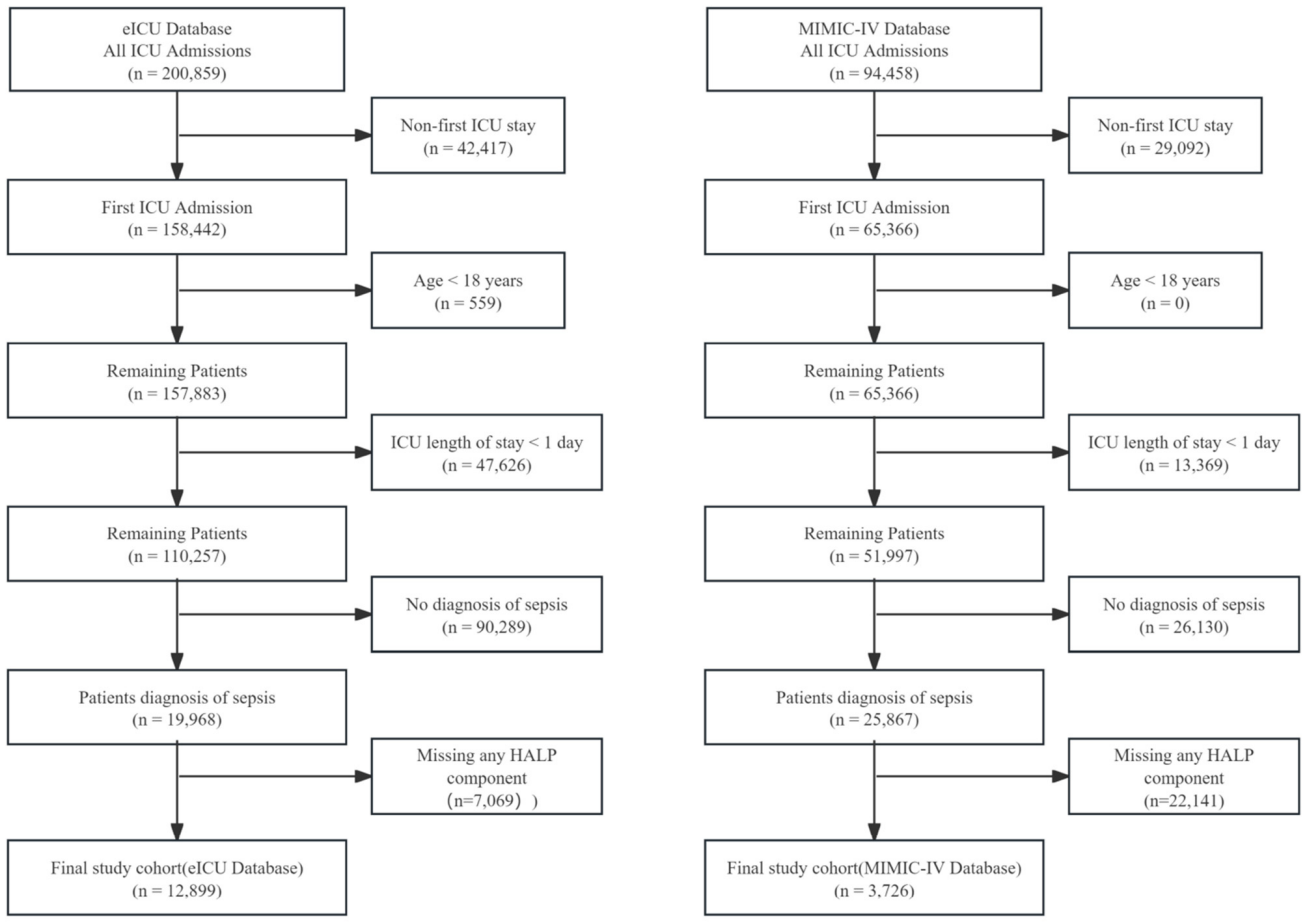
Flow chart of patient selection from the eICU Collaborative Research Database and the MIMIC-IV database. After application of all exclusion criteria, 12,899 patients from the eICU database and 3,726 patients from the MIMIC-IV database were included in the final analysis. HALP, hemoglobin, albumin, lymphocyte, and platelet; ICU, intensive care unit.

A total of 16,625 sepsis patients were included in this retrospective cohort study, comprising 12,899 patients from the eICU database and 3,726 patients from the MIMIC-IV database (Figure 1). The baseline characteristics of patients stratified by HALP score groups are presented in Table 1.

**Table 1:** Baseline characteristics of sepsis patients stratified by HALP score groups.

**Characteristic**	**eICU Database (*****n*** = **12,899)**			**MIMIC-IV Database (*****n*** = **3,726)**		
	**Low (*****n*** = **6,447)**	**High (*****n*** = **6,448)**	***P***	**Low (n** = **1,336)**	**High (*****n*** = **2,390)**	***P***
**Demographics**						
Age, M (Q1, Q3)	68.00 (57.00, 78.00)	65.00 (54.00, 77.00)	<0.001	69.00 (59.00, 79.00)	66.00 (54.00, 76.00)	<0.001
Gender, *n* (%)			0.005			0.023
Male	3,259 (50.55)	3,420 (53.04)		725 (54.27)	1,389 (58.12)	
Female	3,188 (49.45)	3,028 (46.96)		611 (45.73)	1,001 (41.88)	
BMI, M (Q1, Q3)	26.68 (22.51, 32.54)	28.14 (23.77, 34.16)	<0.001	27.10 (23.03, 31.93)	27.97 (24.00, 32.94)	<0.001
**Laboratory parameters**						
WBC, × 10^9^/L, M (Q1, Q3)	12.80 (8.20, 18.50)	13.30 (8.90, 19.00)	<0.001	12.60 (8.20, 18.60)	12.30 (8.60, 17.50)	0.996
Lymphocyte count, × 10^9^/L, M (Q1, Q3)	0.58 (0.35, 0.92)	1.26 (0.86, 1.86)	<0.001	0.53 (0.32, 0.86)	1.32 (0.89, 1.90)	<0.001
Platelet count, × 10^9^/L, M (Q1, Q3)	216.00 (155.50, 298.00)	156.00 (103.00, 214.00)	<0.001	210.50 (142.75, 295.00)	153.00 (99.00, 211.00)	<0.001
Hemoglobin, g/dL, M (Q1, Q3)	9.70 (8.50, 11.10)	10.90 (9.50, 12.40)	<0.001	9.30 (8.00, 10.70)	10.70 (8.90, 12.50)	<0.001
Albumin, g/dL, M (Q1, Q3)	2.30 (1.90, 2.70)	2.70 (2.30, 3.10)	<0.001	2.70 (2.30, 3.10)	3.10 (2.60, 3.50)	<0.001
Glucose, mg/dL, M (Q1, Q3)	157.00 (112.00, 168.00)	157.00 (113.00, 169.00)	0.207	137.00 (107.00, 180.00)	130.00 (106.00, 171.00)	0.028
Lactate, mmol/L, M (Q1, Q3)	2.49 (1.40, 2.49)	2.49 (1.40, 2.49)	<0.001	2.00 (1.30, 3.00)	2.00 (1.40, 3.00)	0.130
BUN, mg/dL, M (Q1, Q3)	29.00 (17.00, 48.00)	27.00 (16.00, 45.00)	<0.001	26.00 (16.00, 45.25)	21.00 (14.00, 36.00)	<0.001
Creatinine, mg/dL, M (Q1, Q3)	1.35 (0.85, 2.40)	1.37 (0.89, 2.40)	0.192	1.20 (0.80, 2.10)	1.10 (0.80, 1.70)	<0.001
INR, M (Q1, Q3)	1.75 (1.30, 1.75)	1.75 (1.30, 1.75)	0.628	1.40 (1.20, 1.60)	1.30 (1.20, 1.60)	0.155
HALP, M (Q1, Q3)	6.57 (4.08, 9.28)	22.77 (16.39, 34.21)	<0.001	7.15 (4.38, 9.70)	27.76 (18.60, 43.04)	<0.001
**Severity scores**						
APS III score, M (Q1, Q3)	73.00 (61.00, 84.00)	73.00 (56.00, 81.00)	<0.001	54.00 (42.00, 68.00)	48.00 (36.00, 64.00)	<0.001
SAPSII, M (Q1, Q3)	60.00 (47.00, 69.00)	60.00 (44.00, 66.00)	<0.001	42.00 (34.00, 53.25)	39.00 (31.00, 48.00)	<0.001
GCS, M (Q1, Q3)	12.00 (12.00, 15.00)	12.00 (11.00, 15.00)	0.021	15.00 (14.00, 15.00)	15.00 (14.00, 15.00)	0.237
**Comorbidities**						
Hypertension, *n* (%)			0.134			0.023
No	5,651 (87.65)	5,595 (86.77)		923 (69.09)	1,564 (65.44)	
Yes	796 (12.35)	853 (13.23)		413 (30.91)	826 (34.56)	
Diabetes mellitus, *n* (%)			0.003			0.226
No	6,194 (96.08)	6,124 (94.98)		916 (68.56)	1,684 (70.46)	
Yes	253 (3.92)	324 (5.02)		420 (31.44)	706 (29.54)	
In-hospital mortality, *n* (%)			<0.001			<0.001
No	5,298 (82.18)	5,547 (86.03)		983 (73.58)	1,921 (80.38)	
Yes	1,149 (17.82)	901 (13.97)		353 (26.42)	469 (19.62)	
ICU mortality, *n* (%)			<0.001			0.012
No	5,761 (89.36)	5,886 (91.28)		1,129 (84.51)	2,090 (87.45)	
Yes	686 (10.64)	562 (8.72)		207 (15.49)	300 (12.55)	
Ventilation, *n* (%)			<0.001			0.693
No	4,272 (66.26)	4,467 (69.28)		172 (12.87)	297 (12.43)	
Yes	2,175 (33.74)	1,981 (30.72)		1,164 (87.13)	2,093 (87.57)	
Hospital length of stay, days, M (Q1, Q3)	9.26 (5.79, 15.74)	8.13 (5.00, 13.92)	<0.001	14.75 (8.77, 24.63)	14.27 (7.96, 24.91)	0.411
ICU length of stay, days, M (Q1, Q3)	3.60 (2.04, 6.80)	3.09 (1.91, 5.98)	<0.001	4.43 (2.45, 8.97)	4.92 (2.50, 10.32)	0.036

Data presented as median (IQR) for continuous variables and *n* (%) for categorical variables. *P*-values calculated using Mann-Whitney U test (continuous) or chi-square test (categorical).

In the eICU database, patients were divided into two groups based on the optimal HALP cutoff value identified by restricted cubic splines analysis, with an inflection point at 12.4548. The low HALP group (≤12.4548) included 6,447 patients (50.0%), while the high HALP group (>12.4548) included 6,448 patients (50.0%). Patients in the low HALP group were significantly older than those in the high HALP group (median age: 68.00 [IQR: 57.00–78.00] vs. 65.00 [IQR: 54.00–77.00] years, p < 0.001). The distribution of gender showed a slight but statistically significant difference between groups (p=0.005), with males comprising 50.55% of the low HALP group and 53.04% of the high HALP group. Body mass index was significantly lower in the low HALP group compared to the high HALP group (26.68 [IQR: 22.51–32.54] vs. 28.14 [IQR: 23.77–34.16] kg/m^2^, *p* < 0.001).

In the MIMIC-IV database, the RCS-derived cutoff was highly consistent, with an inflection point at 12.4551. Based on this threshold, 1,336 patients (35.9%) were classified into the low HALP group (≤12.4551), while 2,390 patients (64.1%) were in the high HALP group (>12.4551). Similar to the eICU cohort, patients in the low HALP group were significantly older (median age: 69.00 [IQR: 59.00–79.00] vs. 66.00 [IQR: 54.00–76.00] years, *p* < 0.001). The gender distribution also differed significantly (*p* = 0.023), with males representing 54.27% of the low HALP group and 58.12% of the high HALP group. BMI was significantly lower in the low HALP group (27.10 [IQR: 23.03–31.93] vs. 27.97 [IQR: 24.00–32.94] kg/m^2^, *p* < 0.001).

### 3.2 Laboratory parameters and HALP score components

#### 3.2.1 eICU database

The laboratory parameters revealed significant differences between HALP groups that aligned with the score calculation methodology. Lymphocyte count was markedly lower in the low HALP group compared to the high HALP group (0.58 [IQR: 0.35–0.92] vs. 1.26 [IQR: 0.86–1.86] × 10^9^/L, *p* < 0.001). Platelet count was significantly higher in the low HALP group (216.00 [IQR: 155.50–298.00] vs. 156.00 [IQR: 103.00–214.00] × 10^9^/L, *p* < 0.001). Hemoglobin levels were lower in the low HALP group (9.70 [IQR: 8.50–11.10] vs. 10.90 [IQR: 9.50-12.40] g/dL, *p* < 0.001), as were albumin levels (2.30 [IQR: 1.90–2.70] vs. 2.70 [IQR: 2.30–3.10] g/dL, *p* < 0.001).

The median HALP score was 6.57 (IQR: 4.08–9.28) in the low HALP group and 22.77 (IQR: 16.39–34.21) in the high HALP group (*p* < 0.001). Other laboratory parameters showed significant differences, including higher BUN levels in the low HALP group (29.00 [IQR: 17.00–48.00] vs. 27.00 [IQR: 16.00–45.00] mg/dL, *p* < 0.001) and higher lactate levels (2.49 [IQR: 1.40–2.49] vs. 2.49 [IQR: 1.40–2.49] mmol/L, *p* < 0.001).

#### 3.2.2 MIMIC-IV database

Similar patterns were observed in the MIMIC-IV cohort. Lymphocyte count was significantly lower in the low HALP group (0.53 [IQR: 0.32–0.86] vs. 1.32 [IQR: 0.89–1.90] × 10^9^/L, *p* < 0.001), while platelet count was higher (210.50 [IQR: 142.75–295.00] vs. 153.00 [IQR: 99.00–211.00] × 10^9^/L, *p* < 0.001). Hemoglobin (9.30 [IQR: 8.00–10.70] vs. 10.70 [IQR: 8.90–12.50] g/dL, *p* < 0.001) and albumin (2.70 [IQR: 2.30–3.10] vs. 3.10 [IQR: 2.60–3.50] g/dL, *p* < 0.001) levels were both significantly lower in the low HALP group.

The median HALP score was 7.15 (IQR: 4.38–9.70) in the low HALP group and 27.76 (IQR: 18.60–43.04) in the high HALP group (*p* < 0.001). BUN levels were significantly higher in the low HALP group (26.00 [IQR: 16.00–45.25] vs. 21.00 [IQR: 14.00–36.00] mg/dL, *p* < 0.001), while creatinine levels were also higher (1.20 [IQR: 0.80–2.10] vs. 1.10 [IQR: 0.80–1.70] mg/dL, *p* < 0.001).

### 3.3 Severity scores and clinical outcomes

#### 3.3.1 Disease severity assessment

In the eICU database, patients in the low HALP group had significantly higher disease severity scores. The APS III score was higher in the low HALP group (73.00 [IQR: 61.00–84.00] vs. 73.00 [IQR: 56.00-81.00], *p* < 0.001), as was the SAPS II score (60.00 [IQR: 47.00–69.00] vs. 60.00 [IQR: 44.00–66.00], *p* < 0.001). Glasgow Coma Scale score were similar between groups (12.00 [IQR: 12.00–15.00] vs. 12.00 [IQR: 11.00–15.00], *p* = 0.021).

In the MIMIC-IV database, the low HALP group demonstrated higher APS III scores (54.00 [IQR: 42.00–68.00] vs. 48.00 [IQR: 36.00-64.00], *p* < 0.001) and SAPS II scores (42.00 [IQR: 34.00–53.25] vs. 39.00 [IQR: 31.00–48.00], p < 0.001). GCS scores showed no significant difference between groups (15.00 [IQR: 14.00–15.00] vs. 15.00 [IQR: 14.00–15.00], *p* = 0.237).

#### 3.3.2 Clinical outcomes

##### 3.3.2.1 Mortality analysis

The primary outcome of in-hospital mortality showed significant differences between HALP groups in both databases. In the eICU database, the low HALP group had a higher in-hospital mortality rate compared to the high HALP group (17.82% vs. 13.97%, *p* < 0.001). Similarly, ICU mortality was higher in the low HALP group (10.64% vs. 8.72%, *p* < 0.001).

In the MIMIC-IV database, the low HALP group demonstrated higher in-hospital mortality (26.42% vs. 19.62%, *p* < 0.001) and ICU mortality (15.49% vs. 12.55%, *p* = 0.012).

##### 3.3.2.2 Healthcare resource utilization

Hospital length of stay was significantly longer in the low HALP group compared to the high HALP group in the eICU database (9.26 [IQR: 5.79–15.74] vs. 8.13 [IQR: 5.00–13.92] days, *p* < 0.001). ICU length of stay was also longer in the low HALP group (3.60 [IQR: 2.04–6.80] vs. 3.09 [IQR: 1.91–5.98] days, *p* < 0.001).

In the MIMIC-IV database, both hospital length of stay (14.75 [IQR: 8.77–24.63] vs. 14.27 [IQR: 7.96–24.91] days, *p* = 0.411) and ICU length of stay (4.43 [IQR: 2.45–8.97] vs. 4.92 [IQR: 2.50–10.32] days, *p* = 0.036) showed differences, with the low HALP group having slightly shorter ICU stays but no significant difference in hospital stays.

### 3.4 Restricted cubic splines analysis

**Figure 2:**
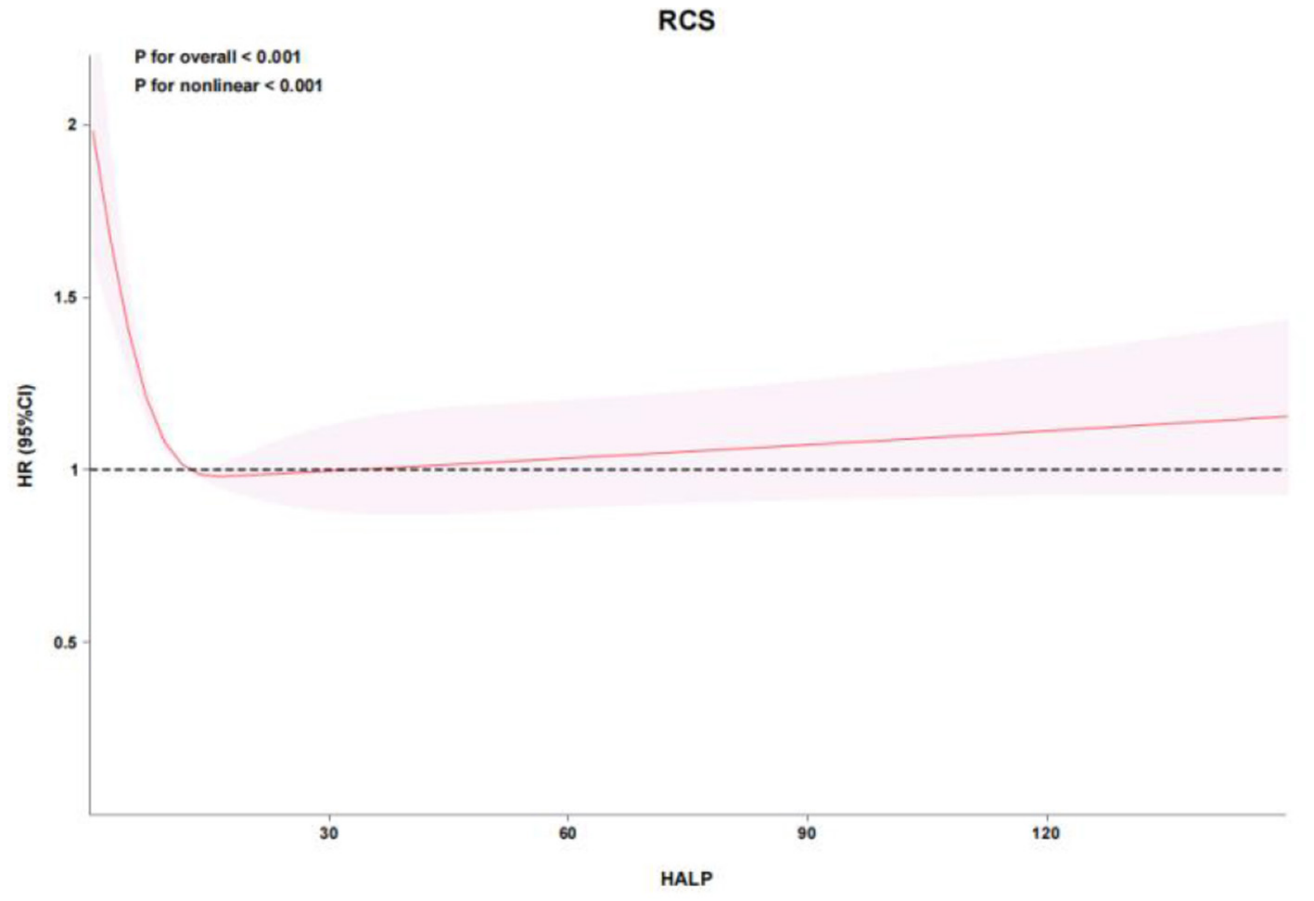
Restricted cubic spline (RCS) analysis of the association between HALP score and hazard ratio for in-hospital mortality in the eICU cohort. A significant nonlinear relationship was observed (P for overall association < 0.001; *P* for nonlinearity < 0.001). The inflection point was approximately 12.4548, below which mortality risk increased sharply.

**Figure 3:**
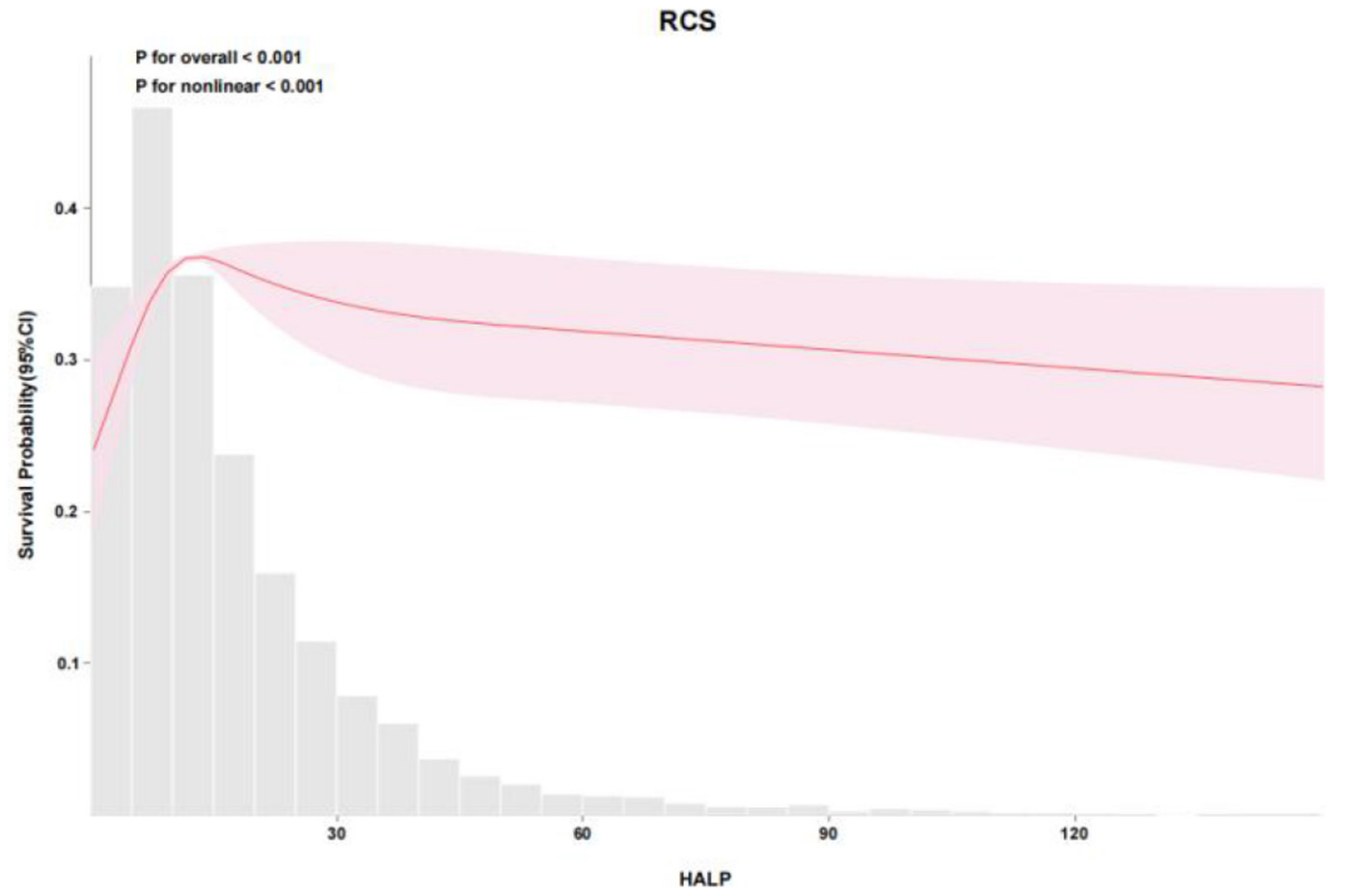
Restricted cubic spline (RCS) analysis of the association between HALP score and survival probability in the eICU cohort. A nonlinear dose–response relationship was observed with an inflection point near 12.4548 (*P* < 0.001).

**Figure 4:**
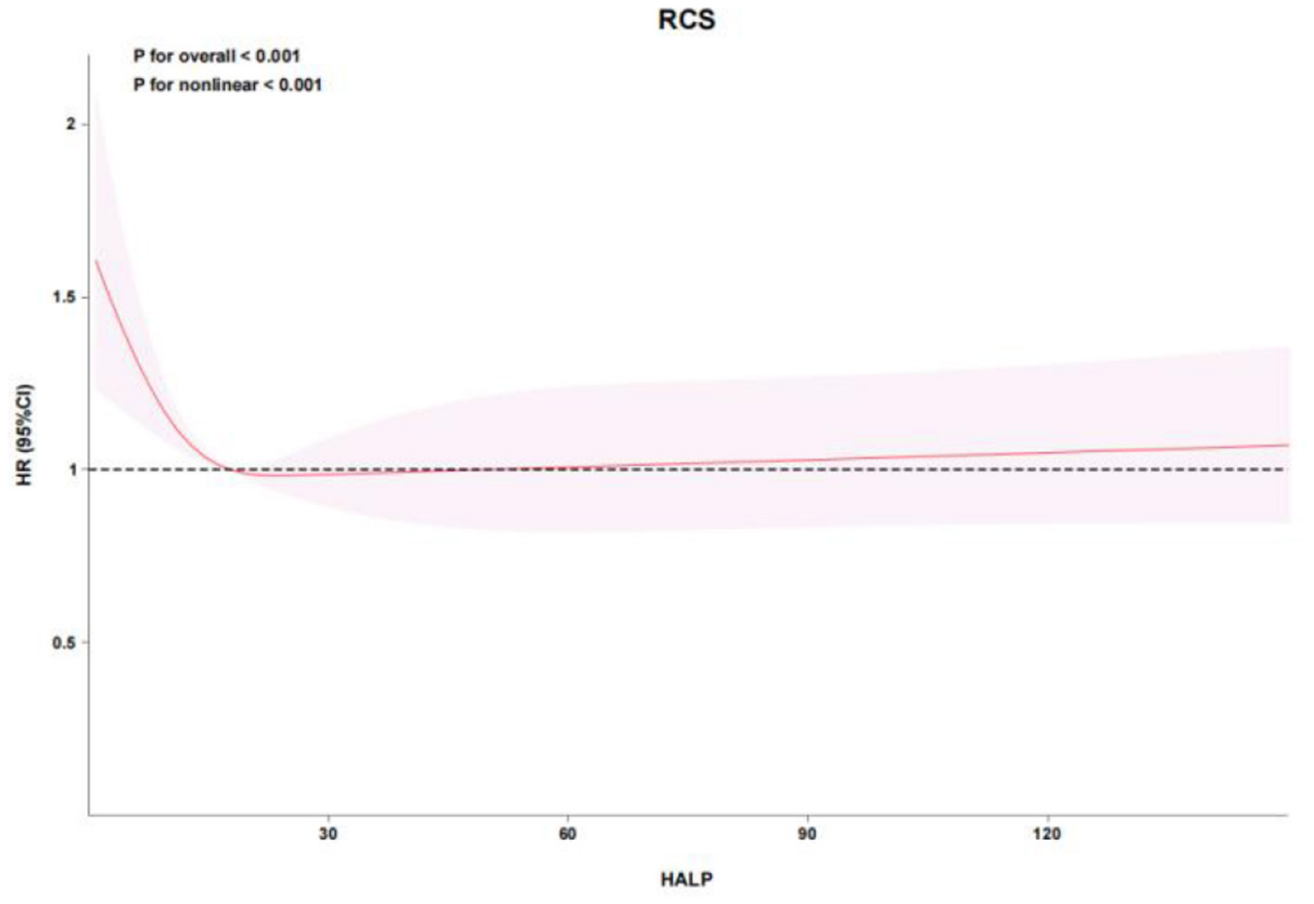
Restricted cubic spline (RCS) analysis of the association between HALP score and hazard ratio for in-hospital mortality in the MIMIC-IV cohort. A significant nonlinear association was identified (*P* for overall association < 0.001; *P* for nonlinearity = 0.002), with an inflection point of approximately 12.4551.

**Figure 5:**
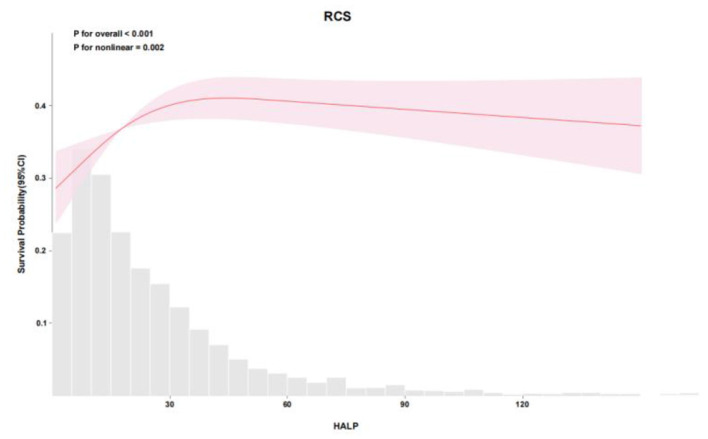
Restricted cubic spline (RCS) analysis of the association between HALP score and survival probability in the MIMIC-IV cohort. Mortality risk rose steeply below the inflection point of 12.4551 (*P* < 0.001).

RCS analysis demonstrated a nonlinear relationship between HALP score and mortality in both cohorts. In the eICU cohort, the adjusted RCS model showed a significant nonlinear association (*P* for overall < 0.001, *P* for nonlinear < 0.001), with mortality risk increasing sharply below a HALP score of approximately 12.4548 (Figures 2, 3). Similarly, in the MIMIC-IV cohort, RCS analysis confirmed a nonlinear relationship (P for overall < 0.001, *P* for nonlinear = 0.002), with a marked increase in mortality risk below a HALP score of approximately 12.4551 (Figures 4, 5). Lower HALP scores were consistently associated with higher mortality risk after adjustment for covariates in both databases.

### 3.5 Kaplan-Meier survival analysis

**Figure 6:**
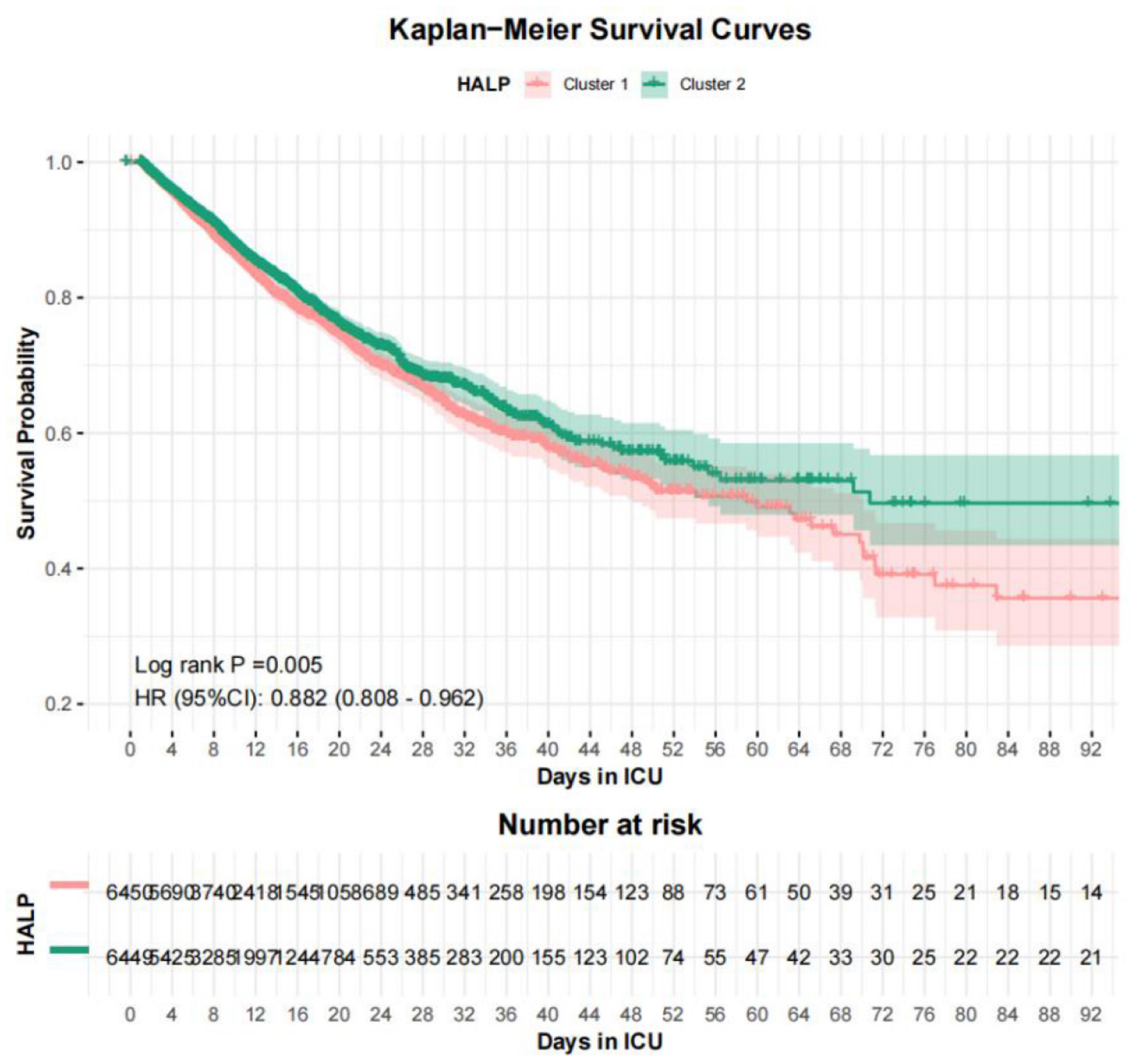
Kaplan–Meier survival curves comparing high and low HALP score groups in the eICU cohort. Patients with higher HALP scores demonstrated significantly improved survival (Log-rank *P* = 0.005). The high HALP group was associated with reduced mortality risk (HR 0.882, 95% CI: 0.808–0.962).

**Figure 7:**
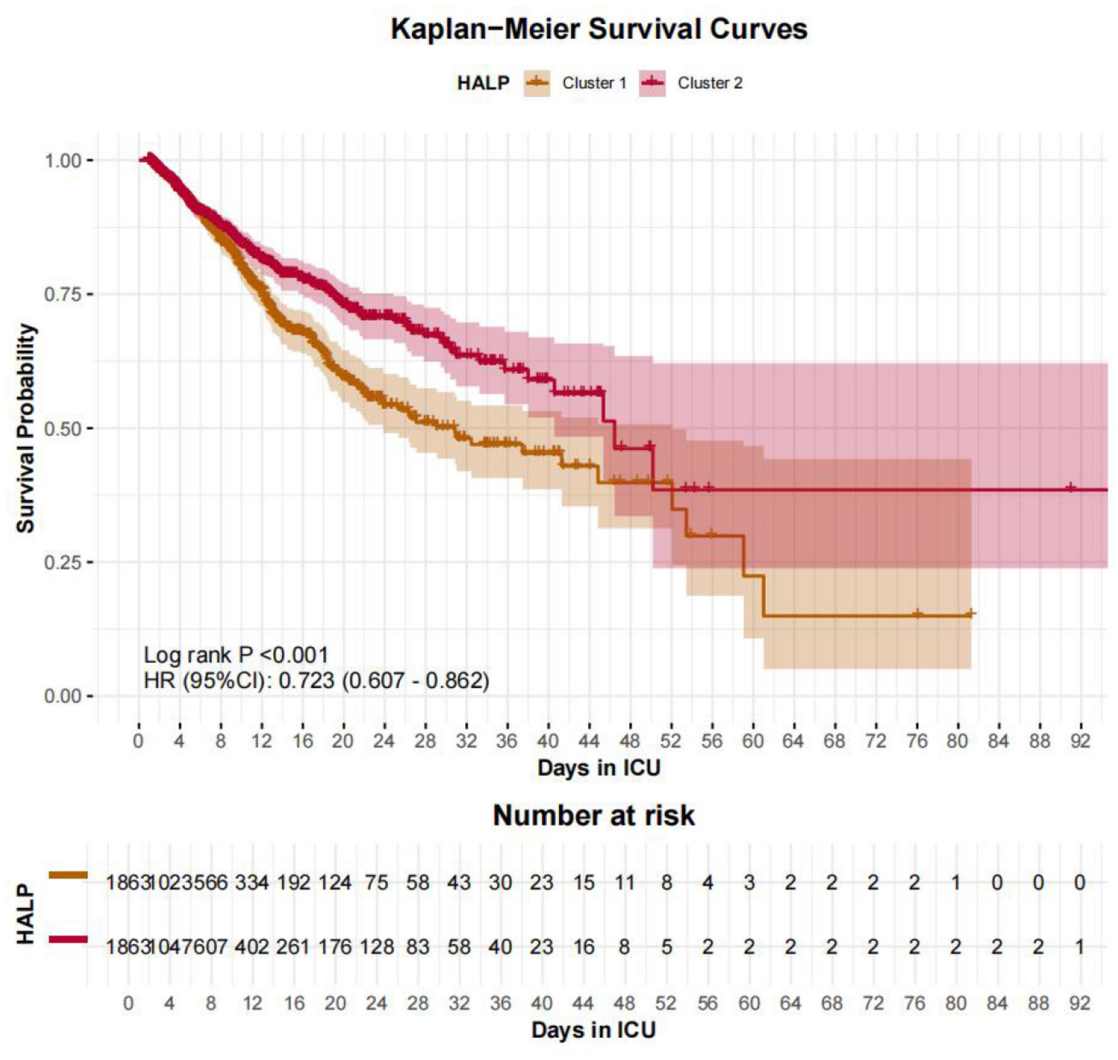
Kaplan–Meier survival curves comparing high and low HALP score groups in the MIMIC-IV cohort. Patients with higher HALP scores had significantly better survival (Log-rank *P* < 0.001). The high HALP group showed a lower hazard of in-hospital mortality (HR 0.723, 95% CI: 0.607–0.862).

Kaplan-Meier survival curves, stratified by RCS-derived HALP cutpoints, revealed significant differences in survival probabilities. In the eICU cohort, the low HALP group (≤12.4548) had worse survival compared to the high HALP group (>12.4548) (Log-rank *P* = 0.005, HR 0.882, 95% CI: 0.808–0.962) (Figure 6). In the MIMIC-IV cohort, the low HALP group (≤12.4551) exhibited significantly worse survival compared to the high HALP group (>12.4551) with stronger discrimination (Log-rank *P* < 0.001, HR 0.723, 95% CI: 0.607–0.862) (Figure 7).

### 3.6 Multivariable cox regression analysis

To further assess the independent association between HALP score groups and in-hospital mortality, multivariable Cox proportional hazards regression was performed using a multi-model strategy in both databases. The results are presented in Table 2 for the eICU database and Table 3 for the MIMIC-IV database.

**Table 2:** Univariable and multivariable cox regression analysis results for HALP score groups in the eICU database.

**Variables**	**Model 1**		**Model 2**		**Model 3**		**Model 4**	
	**HR (95%CI)**	***P***	**HR (95%CI)**	***P***	**HR (95%CI)**	***P***	**HR (95%CI)**	***P***
**HALP custom**								
1	1.00 (Reference)		1.00 (Reference)		1.00 (Reference)		1.00 (Reference)	
2	0.89 (0.81–0.97)	0.007	0.89 (0.81–0.97)	0.008	0.88 (0.80–0.96)	0.003	0.90 (0.82– 0.98)	0.017

HR, Hazard Ratio, CI, Confidence Interval.

Model 1: Crude.

Model 2: Adjust: Age, Gender, Hypertension, Diabetes.

Model 3: Adjust: Age, Gender, Hypertension, Diabetes, WBC, Glucose.

Model 4: Adjust: Age, Gender, Hypertension, Diabetes, WBC, Glucose, Lactate, BUN, Scr, INR, PT, APSIII.

**Table 3:** Univariable and multivariable cox regression analysis results for HALP score groups in the MIMIC-IV database.

**Variables**	**Model 1**		**Model 2**		**Model 3**		**Model 4**	
	**HR (95%CI)**	***P***	**HR (95%CI)**	***P***	**HR (95%CI)**	***P***	**HR (95%CI)**	***P***
**HALP custom**								
1	1.00 (Reference)		1.00 (Reference)		1.00 (Reference)		1.00 (Reference)	
2	0.75 (0.65–0.86)	<0.001	0.79 (0.69–0.91)	<0.001	0.79 (0.69–0.91)	<0.001	0.85 (0.74–0.98)	0.028

HR, Hazard Ratio, CI, Confidence Interval.

Model 1: Crude.

Model 2: Adjust: Age, Gender, Hypertension, Diabetes.

Model 3: Adjust: Age, Gender, Hypertension, Diabetes, WBC, Glucose.

Model 4: Adjust: Age, Gender, Hypertension, Diabetes, WBC, Glucose, Lactate, BUN, Scr, INR, PT, APSIII.

In the eICU database (Table 2), the unadjusted Model 1 showed that the high HALP group had a reduced hazard of mortality compared to the low HALP group (HR 0.89, 95% CI: 0.81-0.97, P=0.007). This association persisted after adjustment for demographics and comorbidities in Model 2 (HR 0.89, 95% CI: 0.81–0.97, *P* = 0.008), further adjustment for white blood cell count and glucose in Model 3 (HR 0.88, 95% CI: 0.80–0.96, *P* = 0.003), and full adjustment in Model 4 (HR 0.90, 95% CI: 0.82–0.98, *P* = 0.017).

In the MIMIC-IV database (Table 3), the unadjusted Model 1 indicated a stronger protective effect for the high HALP group (HR 0.75, 95% CI: 0.65–0.86, *P* < 0.001). After adjustments in Model 2 (HR 0.79, 95% CI: 0.69–0.91, *P* < 0.001), Model 3 (HR 0.79, 95% CI: 0.69–0.91, *P* < 0.001), and full adjustment in Model 4 (HR 0.85, 95% CI: 0.74–0.98, *P* = 0.028), the association remained significant, confirming the independent prognostic value of HALP score.

These findings reinforce the Kaplan-Meier results and demonstrate that the HALP score's association with reduced mortality risk is robust to multivariable adjustment.

### 3.7 Segmented cox proportional hazards analysis

To further explore the dose-response relationship, segmented Cox proportional hazards models were applied, treating HALP as a continuous variable stratified below and above the identified thresholds (12.4548 for eICU and 12.4551 for MIMIC-IV). Models were adjusted for age, sex, white blood cell count, glucose, lactate, international normalized ratio, blood urea nitrogen, serum creatinine, hypertension, diabetes, and APS III.

In the eICU database, below the threshold of 12.4548, each unit increase in HALP score was associated with a 3% reduction in mortality risk (HR 0.97, 95% CI: 0.95–0.99, *P* = 0.002). Above the threshold, the association was not significant (HR 1.00, 95% CI: 0.99–1.01, *P* = 0.541) (Supplementary Tables S1, S2).

In the MIMIC-IV database, similar patterns were observed: below the threshold of 12.4551, each unit increase in HALP was linked to a 3% mortality reduction (HR 0.97, 95% CI: 0.94–0.99, *P* = 0.008), while above the threshold, no significant association was found (HR 1.00, 95% CI: 0.99–1.01, *P* = 0.672) (Supplementary Tables S3, S4).

Multicollinearity diagnostics showed no evidence of linear dependence between HALP and other covariates, with all VIFs < 2 and all pairwise correlations |r| < 0.10 in both databases (Supplementary Figures S1–S4).

### 3.8 Statistical analysis

The HALP score itself satisfied the proportional hazards assumption in both the derivation and validation cohorts (eICU: χ^2^ = 2.14, *df* = 1, *P* = 0.144; MIMIC-IV: χ^2^ = 0.88, *df* = 1, *P* = 0.349; Supplementary Table S5). Although global tests were significant due to known time-varying covariates (primarily lactate and severity scores), sensitivity analyses using lactate tertile stratification confirmed the robustness of the independent protective effect of high HALP (eICU: HR 0.94, 95% CI 0.86–1.02, *P* = 0.135; MIMIC-IV: HR 0.87, 95% CI 0.75–0.99, *P* = 0.044; Supplementary Table S6). Competing-risk analysis using the Fine-Gray model, treating live discharge as a competing event, further confirmed the protective association (eICU: subdistribution HR 0.915, 95% CI 0.835–1.002, *P* = 0.057; MIMIC-IV: subdistribution HR 0.878, 95% CI 0.758–1.017, *P* = 0.082; Supplementary Table S7). Scaled Schoenfeld residual plots for HALP and log-minus-log survival curves by HALP group demonstrated no systematic trends and essential parallelism in both cohorts (Supplementary Figures S5–S8).

## 4 Discussion

In this large, two-cohort retrospective analysis, we found that the hemoglobin–albumin–lymphocyte–platelet (HALP) score, calculated from routine admission laboratory tests, was strongly and non-linearly associated with in-hospital mortality in patients meeting Sepsis-3 criteria. In both the derivation (eICU-CRD) and external validation (MIMIC-IV) cohorts, lower HALP scores were independently associated with greater mortality risk, and restricted cubic spline modeling identified similar inflection points (~12.45) below which risk rose sharply. This association persisted after multivariable adjustment for demographic factors, comorbidities, key laboratory parameters, and APS III score. While these findings support the HALP score as a potentially useful prognostic tool, it should be viewed as complementary to, rather than replacement for, existing sepsis assessment methods.

Our findings are consistent with and extend prior work showing the prognostic value of HALP in other acute and chronic disease settings. Low HALP has been associated with poor survival in gastrointestinal and urological malignancies (9, 18, 19), cardiovascular disease (20), and frail older populations (21). In the critical care setting, a recent MIMIC-IV analysis of septic patients reported that higher HALP scores were linked to reduced 28-day, 90-day, and 1-year mortality, with a break-point around 24.7 (22). Our study adds to this literature by demonstrating a lower optimal cut-off in an ICU sepsis population, potentially reflecting more profound immune-nutritional compromise and coagulation derangement at presentation.

The nonlinear relationship between HALP score and mortality aligns with the complex pathophysiology of sepsis, where multiple organ systems fail in a cascading manner (23). The sharp increase in mortality risk below the threshold of 12.45 likely represents a critical point where the combined deficits in oxygen delivery (hemoglobin), inflammatory response and nutritional status (albumin), immune function (lymphocytes), and hemostatic capacity (platelets) overwhelm physiological compensatory mechanisms (24). The segmented Cox regression analysis revealed an interesting pattern: below the threshold, each unit increase in HALP score was associated with a 3% reduction in mortality risk, while above the threshold, the relationship became less pronounced or even reversed in the eICU cohort. This finding suggests that the HALP score's prognostic value is most relevant for identifying high-risk patients rather than stratifying those with relatively preserved physiological parameters. The potential increase in risk at very high HALP scores observed in the eICU cohort warrants cautious interpretation and may reflect unmeasured confounders such as reactive thrombocytosis or hemoconcentration states (25).

Each component of the HALP score reflects established pathophysiological processes in sepsis. Anemia in sepsis results from multiple mechanisms including suppressed erythropoiesis, increased destruction, and dilution from resuscitation fluids, with hemoglobin levels below 7–9 g/dL associated with increased mortality (26). Hypoalbuminemia, occurring in up to 40–50% of sepsis patients, reflects not only malnutrition but also increased vascular permeability, decreased hepatic synthesis, and consumption during the acute phase response (27). Lymphopenia, present in approximately 60% of sepsis patients, indicates immunosuppression and has been linked to increased secondary infections and mortality (28). Thrombocytopenia, while traditionally associated with poor outcomes, shows a more complex relationship when integrated into the HALP formula, where its position in the denominator accounts for consumption coagulopathy (29).

The integration of these four parameters into a single composite score may better reflect synergistic pathophysiological interactions that could be overlooked when considering individual markers in isolation. Nonetheless, the HALP score represents a simplification of complex biological processes and may not fully capture the heterogeneity of sepsis. Distinct sepsis phenotypes, such as those predominantly characterized by immunosuppression or those driven by hyperinflammation, may exhibit differential associations with the individual components of the HALP score (30).

Our findings build on growing evidence of immunometabolic dysregulation as a key driver of poor outcomes in sepsis and trauma, where simple biomarkers can aid early risk stratification. Building on this, Xu et al. developed a nomogram for persistent inflammation, immunosuppression, and catabolism syndrome (PIICS) in severe trauma (*n* = 215), using LASSO-selected variables like SOFA and APACHE II to achieve robust validation (AUC 0.84), emphasizing composite severity markers' prognostic role (31). Similarly, Wang et al. further explored PBMC pyroptosis as a biomarker of ongoing inflammation and immunosuppression in sepsis, linking it to disease progression and long-term outcomes in prospective cohorts (32, 33).

Compared to established severity scores, the HALP score offers simplicity but potentially less comprehensive assessment. The SOFA score evaluates six organ systems and has been validated extensively for sepsis prognostication and organ dysfunction monitoring. APACHE II incorporates physiological variables, age, and chronic health status, providing broader patient assessment. The HALP score should not replace these validated tools but might serve as a rapid screening instrument, particularly in resource-limited settings where comprehensive severity scoring is impractical.

In contrast, this multicenter analysis (*n* = 16,625) offers superior scale and rigor through external validation in an independent database, explicit nonlinear restricted cubic spline modeling to uncover data-driven thresholds, and comprehensive sensitivity analyses (including proportional hazards testing, lactate stratification, and competing-risk modeling).

Several limitations should be acknowledged. First, the retrospective design precludes causal inference, and the requirement for complete HALP components along with the exclusion of ICU stays shorter than 24 h may introduce selection bias. Although we adjusted for a wide range of covariates, residual confounding from unmeasured clinical factors such as fluid balance, transfusion practices, nutritional support, infection site, microbiological findings, and vasopressor use cannot be fully excluded. The use of worst laboratory values within the first 24 h reflects early illness severity but may not represent the optimal prognostic window. In addition, the two databases originate from United States health systems and cover different practice eras, which may influence generalizability and highlights the need for validation in more diverse international settings.

Beyond these methodological considerations, the application of HALP to sepsis requires additional clinical context. Although HALP was originally developed in oncology, its components collectively capture early immuno-nutritional reserves that remain relevant in sepsis, despite the rapidly evolving inflammatory and hematologic shifts characteristic of critical illness. In this context, HALP should be interpreted as a pragmatic summary marker rather than a mechanism-specific signal, and patients with low HALP may warrant closer monitoring of nutritional and hematologic deterioration. Future prospective studies are needed to validate thresholds, assess HALP trajectories, and explore interactions with nutritional, immunologic, or transfusion-related interventions.

## 5 Conclusion

The HALP score represents a convenient and potentially useful tool for prognostic assessment in sepsis, combining hemoglobin, albumin, lymphocyte, and platelet levels into a single, easily obtainable index. By reflecting both nutritional and immune status, it may provide an additional perspective to support early risk stratification and inform clinical decision making.

## Data Availability

Publicly available datasets were analyzed in this study. This data can be found here: https://physionet.org/content/eicu-crd/2.0/; MIMIC-IV: https://physionet.org/content/mimiciv/2.2/.
