# Supplementary material for: Association between HALP score and in-hospital mortality in sepsis patients: a multicenter retrospective cohort study with external validation
Source: Front Public Health. 2026 Jan 12;13:1710118. doi: 10.3389/fpubh.2025.1710118 (PMC12832424; doi:10.3389/fpubh.2025.1710118)
Supplement: Supplementary file 1 [file Supplementary_file_1.pdf]

```sql
-- ******************** 1. eICU-CRD v2.0 ********************
-- Table prefixes in eICU: patient, apachepatientresult, lab, vitalperiodic, etc.

WITH suspected_infection AS (
    -- Antibiotic exposure ±24h of ICU admission + culture taken ±24h (standard Angus criteria adapted)
    SELECT DISTINCT p.patientunitstayid
    FROM patient p
    INNER JOIN medication m ON p.patientunitstayid = m.patientunitstayid
    INNER JOIN nursecharting nc ON p.patientunitstayid = nc.patientunitstayid
    WHERE m.drugname ILIKE ANY(ARRAY['%vancomycin%','%piperacillin%','%tazobactam%','%cefepime%','%ceftazidime%','%meropenem%','%imipenem%','%levofloxacin%','%ciprofloxacin%','%azithromycin%','%ceftriaxone%'])
      AND m.drugstartoffset BETWEEN -1440 AND 8640      -- ±24h relative to ICU admission (offset in minutes)
      AND nc.nursingchartcelltypevallabel ILIKE '%culture%'
      AND nc.nursingchartoffset BETWEEN -1440 AND 8640
),

sofa_components AS (
    SELECT
        l.patientunitstayid,
        -- Respiratory: PaO2/FiO2 (worst in ±24h)
        MIN(CASE WHEN l.labname = 'paO2' THEN l.labresult END /
            GREATEST(0.21, COALESCE(v.fio2/100.0, 0.21))) AS pf_ratio,
        -- Coagulation: platelets ×10^3 / μL
        MIN(l.labresult) AS platelets_min
        FILTER (WHERE l.labname IN ('platelets x 1000')),
        -- Liver: bilirubin (mg/dL)
        MAX(l.labresult) AS bilirubin_max
        FILTER (WHERE l.labname = 'total bilirubin'),
        -- Cardiovascular: mean arterial pressure or vasopressor use
        CASE WHEN MIN(v.systemicmean) < 70 THEN 1
             WHEN EXISTS (SELECT 1 FROM medication m
                          WHERE m.patientunitstayid = l.patientunitstayid
                            AND m.drugname ILIKE ANY(ARRAY['%norepinephrine%','%epinephrine%','%dopamine%','%vasopressin%','%phenylephrine%'])
                            AND m.drugstartoffset BETWEEN -1440 AND 8640) THEN 1
             ELSE 0 END AS cardio_score,
        -- CNS: Glasgow Coma Scale
        MIN(a.gcs) AS gcs_min,
        -- Renal: creatinine (mg/dL)
```

```sql
            MAX(l.labresult) AS creatinine_max
            FILTER (WHERE l.labname = 'creatinine')
        FROM lab l
        LEFT JOIN vitalperiodic v ON l.patientunitstayid = v.patientunitstayid
            AND ABS(l.labresultoffset - v.observationoffset) < 120
        LEFT JOIN apachepatientresult a ON l.patientunitstayid = a.patientunitstayid
        WHERE l.labresultoffset BETWEEN -1440 AND 8640
        GROUP BY l.patientunitstayid
),

sofa_score AS (
        SELECT
            sc.*,
            -- Final SOFA score (standard scoring rules)
            (CASE WHEN pf_ratio < 100 THEN 4 WHEN pf_ratio < 200 THEN 3 WHEN pf_ratio < 300
THEN 2 WHEN pf_ratio < 400 THEN 1 ELSE 0 END) AS resp_score,
            (CASE WHEN platelets_min < 20 THEN 4 WHEN platelets_min < 50 THEN 3 WHEN
platelets_min < 100 THEN 2 WHEN platelets_min < 150 THEN 1 ELSE 0 END) AS coag_score,
            (CASE WHEN bilirubin_max >= 12 THEN 4 WHEN bilirubin_max >= 6 THEN 3 WHEN
bilirubin_max >= 2 THEN 2 WHEN bilirubin_max >= 1.2 THEN 1 ELSE 0 END) AS liver_score,
            (CASE WHEN cardio_score = 1 THEN 1 ELSE 0 END) +
            (CASE WHEN EXISTS (SELECT 1 FROM medication m WHERE m.patientunitstayid =
sc.patientunitstayid
                        AND m.drugname ILIKE '%norepinephrine%' AND
m.drugrate::numeric > 0.1) THEN 3
                  WHEN EXISTS (SELECT 1 FROM medication m WHERE m.patientunitstayid =
sc.patientunitstayid
                        AND m.drugname ILIKE '%dopamine%' AND m.drugrate::numeric > 5)
THEN 2 ELSE 0 END) AS cv_score,
            (CASE WHEN gcs_min < 6 THEN 4 WHEN gcs_min < 10 THEN 3 WHEN gcs_min < 13
THEN 2 WHEN gcs_min < 15 THEN 1 ELSE 0 END) AS cns_score,
            (CASE WHEN creatinine_max >= 5 THEN 4 WHEN creatinine_max >= 3.5 THEN 3 WHEN
creatinine_max >= 2 THEN 2 WHEN creatinine_max >= 1.2 THEN 1 ELSE 0 END) AS renal_score
        FROM sofa_components sc
),

sepsis_cohort_eicu AS (
        SELECT DISTINCT p.patientunitstayid,
               p.hospitaldischargestatus AS deathinhosp,
               p.hospitaldischargeoffset/1440.0 AS hospday,
               (resp_score + coag_score + liver_score + cv_score + cns_score + renal_score) >= 2
AS sepsis3
        FROM patient p
        INNER JOIN suspected_infection si ON p.patientunitstayid = si.patientunitstayid
```

```sql
    INNER JOIN sofa_score ss ON p.patientunitstayid = ss.patientunitstayid
    WHERE (resp_score + coag_score + liver_score + cv_score + cns_score + renal_score) >= 2
),

halp_eicu AS (
    SELECT
        l.patientunitstayid,
        -- Unified units: g/L for Hb & albumin, ×10^9/L for lymph & platelet
        (MAX(CASE WHEN l.labname = 'Hgb' THEN l.labresult * 10 END)
          * MAX(CASE WHEN l.labname = 'albumin' THEN l.labresult * 10 END)
          * MAX(CASE WHEN l.labname = 'lymphocytes' THEN l.labresult END)
        ) / NULLIF(MAX(CASE WHEN l.labname = 'platelets x 1000' THEN l.labresult END), 0) AS HALP
    FROM lab l
    WHERE l.labresultoffset BETWEEN -1440 AND 1440
      AND l.labname IN ('Hgb','albumin','lymphocytes','platelets x 1000')
    GROUP BY l.patientunitstayid
)

-- Final eICU cohort (example, merge with other covariates as needed)
SELECT e.*, h.HALP
FROM sepsis_cohort_eicu e
LEFT JOIN halp_eicu h ON e.patientunitstayid = h.patientunitstayid;


-- ******************** 2. MIMIC-IV v2.2 ********************
-- Core tables: admissions, icustays, labevents, chartevents, inputevents, etc.

WITH suspected_infection AS (
    SELECT DISTINCT icu.subject_id, icu.hadm_id, icu.stay_id
    FROM icustays icu
    INNER JOIN antibiotic_exposure ab ON icu.stay_id = ab.stay_id
    INNER JOIN microbiologyevents me ON icu.hadm_id = me.hadm_id
    WHERE ab.starttime BETWEEN icu.intime - INTERVAL '24 hours' AND icu.intime + INTERVAL '24 hours'
      AND me.charttime BETWEEN icu.intime - INTERVAL '24 hours' AND icu.intime + INTERVAL '24 hours'
),

sofa AS (
    -- Standard MIMIC-IV SOFA calculation (widely used in MIMIC literature)
    SELECT
        ie.subject_id, ie.hadm_id, ie.stay_id,
        -- PaO2/FiO2, platelets, bilirubin, MAP/vasopressors, GCS, creatinine
```

```
        -- (omitted full subquery for brevity; identical to published MIMIC-IV SOFA scripts)
        -- Final SOFA ≥2 flag
        sofa_score >= 2 AS sepsis3
    FROM icustays ie
    LEFT JOIN derived_sofa ds ON ie.stay_id = ds.stay_id      -- using pre-comized derived table
or custom query
),

halp_mimic AS (
    SELECT
        le.subject_id, le.hadm_id, icu.stay_id,
        (MAX(CASE WHEN le.itemid IN (50809,51222) THEN le.valuenum * 10 END)        --
hemoglobin g/dL → g/L
         * MAX(CASE WHEN le.itemid = 50862 THEN le.valuenum * 10 END)               --
albumin g/dL → g/L
         * MAX(CASE WHEN le.itemid = 51244 THEN le.valuenum END)                    --
lymphocyte %
         * 10) / NULLIF(MAX(CASE WHEN le.itemid = 51265 THEN le.valuenum END),0) AS
HALP_abs_lymph
        / NULLIF(MAX(CASE WHEN le.itemid = 51279 THEN le.valuenum END),0) AS HALP      --
platelet
    FROM labevents le
    INNER JOIN icustays icu ON le.hadm_id = icu.hadm_id
    WHERE le.charttime BETWEEN icu.intime - INTERVAL '24 hours' AND icu.intime + INTERVAL
'24 hours'
      AND le.itemid IN (50809,51222,50862,51244,51265,51279)       -- Hb, albumin, lymph,
platelet
    GROUP BY le.subject_id, le.hadm_id, icu.stay_id
)

-- Final MIMIC-IV cohort
SELECT i.subject_id, i.hadm_id, i.stay_id,
       a.hospital_expire_flag AS ishospdead,
       EXTRACT(DAY FROM a.dischtime - a.admittime) AS hospday,
       h.HALP
FROM icustays i
INNER JOIN suspected_infection si ON i.stay_id = si.stay_id
INNER JOIN admissions a ON i.hadm_id = a.hadm_id
INNER JOIN sofa s ON i.stay_id = s.stay_id AND s.sepsis3
LEFT JOIN halp_mimic h ON i.stay_id = h.stay_id;
```
