# Supplementary material for: Association between HALP score and in-hospital mortality in sepsis patients: a multicenter retrospective cohort study with external validation
Source: Front Public Health. 2026 Jan 12;13:1710118. doi: 10.3389/fpubh.2025.1710118 (PMC12832424; doi:10.3389/fpubh.2025.1710118)
Supplement: Supplementary file 2 [file Supplementary_file_2.docx]

**Supplementary Material S2. Variable Dictionary and Definitions**

| **Variable Name** | **Database Source** | **Definition and Unit** | **Notes** |
| --- | --- | --- | --- |
| **Age** | eICU, MIMIC-IV | Age at ICU admission (years) | Continuous variable |
| **Sex** | eICU, MIMIC-IV | Male or female | Binary variable |
| **Heart rate** | eICU, MIMIC-IV | First available value within ±24 h of ICU admission (beats/min) | Vital sign |
| **Mean arterial pressure** | eICU, MIMIC-IV | First available value within ±24 h (mmHg) | Vital sign |
| **Respiratory rate** | eICU, MIMIC-IV | First available value within ±24 h (breaths/min) | Vital sign |
| **Temperature** | eICU, MIMIC-IV | First available value within ±24 h (°C) | Vital sign |
| **Hemoglobin** | eICU, MIMIC-IV | Lowest value within ±24 h (g/L) | Converted to g/L for HALP |
| **Albumin** | eICU, MIMIC-IV | Lowest value within ±24 h (g/L) | Converted to g/L for HALP |
| **Lymphocyte count** | eICU, MIMIC-IV | Lowest value within ±24 h (×10⁹/L) | HALP component |
| **Platelet count** | eICU, MIMIC-IV | Lowest value within ±24 h (×10⁹/L) | HALP component |
| **HALP score** | Derived | Hemoglobin × Albumin × Lymphocyte count ÷ Platelet count | Unit-harmonized composite index |
| **PaO₂/FiO₂ ratio** | eICU, MIMIC-IV | Worst value within ±24 h | SOFA respiratory component |
| **Vasopressor use** | eICU, MIMIC-IV | Any vasopressor within ±24 h | SOFA cardiovascular component |
| **Bilirubin** | eICU, MIMIC-IV | Highest value within ±24 h (mg/dL) | SOFA liver component |
| **Creatinine** | eICU, MIMIC-IV | Highest value within ±24 h (mg/dL) | SOFA renal component |
| **Urine output** | eICU, MIMIC-IV | Total in first 24 h | SOFA renal component |
| **Platelet count (SOFA)** | eICU, MIMIC-IV | Lowest within ±24 h (×10⁹/L) | SOFA coagulation component |
| **Glasgow Coma Scale** | eICU, MIMIC-IV | Lowest within ±24 h | SOFA neurologic component |
| **Mechanical ventilation** | eICU, MIMIC-IV | Any invasive or non-invasive ventilation during ICU stay | Binary |
| **In-hospital mortality** | eICU, MIMIC-IV | ICU or hospital death | Outcome |
