# Supplementary material for: Association between HALP score and in-hospital mortality in sepsis patients: a multicenter retrospective cohort study with external validation
Source: Front Public Health. 2026 Jan 12;13:1710118. doi: 10.3389/fpubh.2025.1710118 (PMC12832424; doi:10.3389/fpubh.2025.1710118)
Supplement: Supplementary file 3 [file Supplementary_file_3.docx]

**Supplementary Material S3. STROBE Checklist for Cohort Studies**

**STROBE Checklist (Cohort Study)**

| **Item No.** | **STROBE Recommendation** | **Section in Manuscript** | **Completed** |
| --- | --- | --- | --- |
| **Title and Abstract** |  |  |  |
| 1 | Indicate the study design in the title or abstract; provide informative and balanced summary | Title, Abstract | Yes |
| **Introduction** |  |  |  |
| 2 | Explain scientific background and rationale | Introduction | Yes |
| 3 | State specific objectives, including hypotheses | Introduction | Yes |
| **Methods** |  |  |  |
| 4 | Present key elements of study design early | Methods: Study Design | Yes |
| 5 | Describe setting, locations, and relevant dates | Methods: Data Sources | Yes |
| 6a | Give eligibility criteria and methods of participant selection | Methods: Study Population | Yes |
| 6b | For matched studies, give matching criteria | Not applicable | N/A |
| 7 | Clearly define all outcomes, exposures, predictors, confounders | Methods: Variables; Supplement S1 | Yes |
| 8 | Provide data sources and measurement details for each variable | Methods; Supplement S1 | Yes |
| 9 | Describe efforts to address potential sources of bias | Methods: Sensitivity Analyses; Discussion | Yes |
| 10 | Explain study size (sample size determination or full-cohort use) | Methods: Study Population | Yes |
| 11 | Explain handling of quantitative variables | Methods: Statistical Analysis | Yes |
| 12a | Describe all statistical methods, including confounding control | Methods: Statistical Analysis | Yes |
| 12b | Methods for subgroups and interactions | Methods: Statistical Analysis | Yes |
| 12c | Explain handling of missing data | Methods; Discussion | Yes |
| 12d | Address loss to follow-up, if applicable | Not applicable | N/A |
| 12e | Sensitivity analyses | Methods; Supplement | Yes |
| **Results** |  |  |  |
| 13a | Report participant numbers at each stage | Results; Figure 1 Flowchart | Yes |
| 13b | Provide reasons for non-participation at each stage | Results; Figure 1 | Yes |
| 13c | Provide a flow diagram | Figure 1 | Yes |
| 14a | Give characteristics of study participants | Table 1 | Yes |
| 14b | Indicate number of participants with missing data | Supplement S3 | Yes |
| 15 | Report outcome events over time | Results | Yes |
| 16a | Provide unadjusted and adjusted estimates with precision | Results; Tables 2–4 | Yes |
| 16b | Report categorization of continuous variables, if applicable | Methods; Results | Yes |
| 16c | If relevant, translate relative risks to absolute risks | Not applicable | N/A |
| **Discussion** |  |  |  |
| 18 | Summarize key results with reference to objectives | Discussion | Yes |
| 19 | Discuss limitations, addressing bias and imprecision | Discussion: Limitations | Yes |
| 20 | Provide cautious overall interpretation | Discussion | Yes |
| 21 | Discuss generalizability of study findings | Discussion | Yes |
| **Other Information** |  |  |  |
| 22 | Funding source and role of funders | Funding Statement | Yes |
