# Supplementary material for: Association between HALP score and in-hospital mortality in sepsis patients: a multicenter retrospective cohort study with external validation
Source: Front Public Health. 2026 Jan 12;13:1710118. doi: 10.3389/fpubh.2025.1710118 (PMC12832424; doi:10.3389/fpubh.2025.1710118)
Supplement: Supplementary file 4 [file Table_1.docx]

| Variables | Univariate | | | | |  | Multivariate | | | | |
| --- | --- | --- | --- | --- | --- | --- | --- | --- | --- | --- | --- |
|  | β | S.E | Z | P | HR (95%CI) |  | β | S.E | Z | P | HR (95%CI) |
| HALP | -0.03 | 0.01 | -3.69 | <.001 | 0.97 (0.95 ~ 0.98) |  | -0.03 | 0.01 | -3.32 | <.001 | 0.97 (0.95 ~ 0.99) |
| Gender |  |  |  |  |  |  |  |  |  |  |  |
| Male |  |  |  |  | 1.00 (Reference) |  |  |  |  |  | 1.00 (Reference) |
| Female | -0.06 | 0.06 | -0.93 | 0.352 | 0.95 (0.84 ~ 1.06) |  | -0.05 | 0.06 | -0.82 | 0.411 | 0.95 (0.85 ~ 1.07) |
| Hypertension |  |  |  |  |  |  |  |  |  |  |  |
| No |  |  |  |  | 1.00 (Reference) |  |  |  |  |  | 1.00 (Reference) |
| Yes | -0.03 | 0.09 | -0.31 | 0.757 | 0.97 (0.82 ~ 1.15) |  | 0.02 | 0.09 | 0.26 | 0.794 | 1.02 (0.86 ~ 1.21) |
| Diabetes mellitus |  |  |  |  |  |  |  |  |  |  |  |
| No |  |  |  |  | 1.00 (Reference) |  |  |  |  |  | 1.00 (Reference) |
| Yes | 0.16 | 0.12 | 1.30 | 0.194 | 1.17 (0.92 ~ 1.50) |  | 0.02 | 0.13 | 0.15 | 0.877 | 1.02 (0.80 ~ 1.31) |
| Age | 0.03 | 0.00 | 12.71 | <.001 | 1.03 (1.02 ~ 1.03) |  | 0.02 | 0.00 | 10.34 | <.001 | 1.02 (1.02 ~ 1.03) |
| WBC | 0.01 | 0.00 | 4.05 | <.001 | 1.01 (1.01 ~ 1.02) |  | 0.01 | 0.00 | 1.97 | 0.049 | 1.01 (1.01 ~ 1.01) |
| Glu | -0.00 | 0.00 | -0.92 | 0.360 | 1.00 (1.00 ~ 1.00) |  | -0.01 | 0.00 | -2.30 | 0.022 | 0.99 (0.99 ~ 0.99) |
| Lactate | 0.13 | 0.01 | 12.80 | <.001 | 1.14 (1.12 ~ 1.17) |  | 0.11 | 0.01 | 8.93 | <.001 | 1.11 (1.09 ~ 1.14) |
| BUN | 0.01 | 0.00 | 6.31 | <.001 | 1.01 (1.01 ~ 1.01) |  | 0.01 | 0.00 | 3.21 | 0.001 | 1.01 (1.01 ~ 1.01) |
| Scr | 0.02 | 0.01 | 1.36 | 0.175 | 1.02 (0.99 ~ 1.05) |  | -0.05 | 0.02 | -2.49 | 0.013 | 0.95 (0.91 ~ 0.99) |
| INR | 0.18 | 0.02 | 9.20 | <.001 | 1.20 (1.15 ~ 1.24) |  | 0.18 | 0.06 | 2.86 | 0.004 | 1.20 (1.06 ~ 1.36) |
| APSIII | 0.02 | 0.00 | 14.70 | <.001 | 1.02 (1.01 ~ 1.02) |  | 0.01 | 0.00 | 8.81 | <.001 | 1.01 (1.01 ~ 1.01) |

**Supplementary Table 1: Association Between HALP Score (as a Continuous Variable) and In-Hospital Mortality in the eICU Cohort: Cox Proportional Hazards Analysis (Left-Side of the Threshold)**
