# Supplementary material for: Association between HALP score and in-hospital mortality in sepsis patients: a multicenter retrospective cohort study with external validation
Source: Front Public Health. 2026 Jan 12;13:1710118. doi: 10.3389/fpubh.2025.1710118 (PMC12832424; doi:10.3389/fpubh.2025.1710118)
Supplement: Supplementary file 5 [file Table_2.docx]

| Variables | Univariate | | | | |  | Multivariate | | | | |
| --- | --- | --- | --- | --- | --- | --- | --- | --- | --- | --- | --- |
|  | β | S.E | Z | P | HR (95%CI) |  | β | S.E | Z | P | HR (95%CI) |
| HALP | 0.01 | 0.00 | 2.29 | 0.022 | 1.01 (1.01 ~ 1.01) |  | 0.01 | 0.00 | 1.98 | 0.047 | 1.01 (1.01 ~ 1.01) |
| Gender |  |  |  |  |  |  |  |  |  |  |  |
| Male |  |  |  |  | 1.00 (Reference) |  |  |  |  |  | 1.00 (Reference) |
| Female | 0.08 | 0.07 | 1.14 | 0.253 | 1.08 (0.95 ~ 1.23) |  | -0.02 | 0.07 | -0.24 | 0.807 | 0.98 (0.86 ~ 1.12) |
| Hypertension |  |  |  |  |  |  |  |  |  |  |  |
| No |  |  |  |  | 1.00 (Reference) |  |  |  |  |  | 1.00 (Reference) |
| Yes | -0.22 | 0.10 | -2.22 | 0.027 | 0.80 (0.66 ~ 0.97) |  | -0.08 | 0.10 | -0.84 | 0.403 | 0.92 (0.76 ~ 1.12) |
| Diabetes mellitus |  |  |  |  |  |  |  |  |  |  |  |
| No |  |  |  |  | 1.00 (Reference) |  |  |  |  |  | 1.00 (Reference) |
| Yes | -0.27 | 0.15 | -1.82 | 0.068 | 0.76 (0.57 ~ 1.02) |  | -0.50 | 0.15 | -3.26 | 0.001 | 0.61 (0.45 ~ 0.82) |
| Age | 0.02 | 0.00 | 8.28 | <.001 | 1.02 (1.01 ~ 1.02) |  | 0.01 | 0.00 | 6.40 | <.001 | 1.01 (1.01 ~ 1.02) |
| WBC | 0.01 | 0.00 | 3.85 | <.001 | 1.01 (1.01 ~ 1.02) |  | 0.00 | 0.00 | 0.54 | 0.591 | 1.00 (1.00 ~ 1.01) |
| Glu | -0.00 | 0.00 | -0.14 | 0.888 | 1.00 (1.00 ~ 1.00) |  | -0.00 | 0.00 | -1.62 | 0.106 | 1.00 (1.00 ~ 1.00) |
| Lactate | 0.12 | 0.01 | 16.49 | <.001 | 1.13 (1.11 ~ 1.15) |  | 0.09 | 0.01 | 9.34 | <.001 | 1.09 (1.07 ~ 1.11) |
| BUN | 0.01 | 0.00 | 7.31 | <.001 | 1.01 (1.01 ~ 1.01) |  | 0.01 | 0.00 | 3.40 | <.001 | 1.01 (1.01 ~ 1.01) |
| Scr | 0.05 | 0.01 | 3.84 | <.001 | 1.06 (1.03 ~ 1.09) |  | -0.06 | 0.02 | -2.67 | 0.008 | 0.94 (0.90 ~ 0.98) |
| INR | 0.18 | 0.02 | 9.59 | <.001 | 1.20 (1.15 ~ 1.24) |  | 0.14 | 0.02 | 6.20 | <.001 | 1.15 (1.10 ~ 1.21) |
| APSIII | 0.02 | 0.00 | 20.32 | <.001 | 1.02 (1.02 ~ 1.03) |  | 0.02 | 0.00 | 14.98 | <.001 | 1.02 (1.02 ~ 1.02) |

**Supplementary Table 2: Association Between HALP Score (as a Continuous Variable) and In-Hospital Mortality in the eICU Cohort: Cox Proportional Hazards Analysis (Right-Side of the Threshold)**
