# Supplementary material for: Association between HALP score and in-hospital mortality in sepsis patients: a multicenter retrospective cohort study with external validation
Source: Front Public Health. 2026 Jan 12;13:1710118. doi: 10.3389/fpubh.2025.1710118 (PMC12832424; doi:10.3389/fpubh.2025.1710118)
Supplement: Supplementary file 6 [file Table_3.docx]

| Variables | Univariate | | | | |  | Multivariate | | | | |
| --- | --- | --- | --- | --- | --- | --- | --- | --- | --- | --- | --- |
|  | β | S.E | Z | P | HR (95%CI) |  | β | S.E | Z | P | HR (95%CI) |
| HALP | -0.04 | 0.02 | -2.51 | 0.012 | 0.96 (0.93 ~ 0.99) |  | -0.04 | 0.02 | -2.07 | 0.039 | 0.97 (0.93 ~ 0.99) |
|  |  |  |  |  |  |  |  |  |  |  |  |
| Gender, |  |  |  |  |  |  |  |  |  |  |  |
| Male |  |  |  |  | 1.00 (Reference) |  |  |  |  |  | 1.00 (Reference) |
| Female | 0.03 | 0.11 | 0.26 | 0.799 | 1.03 (0.83 ~ 1.27) |  | 0.06 | 0.11 | 0.50 | 0.614 | 1.06 (0.85 ~ 1.31) |
| Hypertension |  |  |  |  |  |  |  |  |  |  |  |
| No |  |  |  |  | 1.00 (Reference) |  |  |  |  |  | 1.00 (Reference) |
| Yes | -0.12 | 0.12 | -0.98 | 0.328 | 0.89 (0.71 ~ 1.12) |  | -0.12 | 0.12 | -1.02 | 0.309 | 0.88 (0.70 ~ 1.12) |
| Diabetes mellitus |  |  |  |  |  |  |  |  |  |  |  |
| No |  |  |  |  | 1.00 (Reference) |  |  |  |  |  | 1.00 (Reference) |
| Yes | 0.05 | 0.11 | 0.46 | 0.649 | 1.05 (0.84 ~ 1.32) |  | 0.05 | 0.12 | 0.41 | 0.685 | 1.05 (0.83 ~ 1.33) |
| Age | 0.02 | 0.00 | 4.46 | <.001 | 1.02 (1.01 ~ 1.03) |  | 0.02 | 0.00 | 4.23 | <.001 | 1.02 (1.01 ~ 1.03) |
| WBC | 0.01 | 0.01 | 1.58 | 0.114 | 1.01 (1.00 ~ 1.02) |  | 0.00 | 0.01 | 0.57 | 0.568 | 1.00 (0.99 ~ 1.01) |
| Glu | 0.00 | 0.00 | 0.69 | 0.488 | 1.00 (1.00 ~ 1.00) |  | -0.00 | 0.00 | -1.04 | 0.296 | 1.00 (1.00 ~ 1.00) |
| Lactate | 0.08 | 0.02 | 4.66 | <.001 | 1.08 (1.04 ~ 1.11) |  | 0.05 | 0.02 | 2.54 | 0.011 | 1.05 (1.01 ~ 1.09) |
| BUN | 0.01 | 0.00 | 2.45 | 0.014 | 1.01 (1.01 ~ 1.01) |  | 0.00 | 0.00 | 1.79 | 0.073 | 1.00 (1.00 ~ 1.01) |
| Scr | -0.01 | 0.03 | -0.33 | 0.742 | 0.99 (0.94 ~ 1.04) |  | -0.11 | 0.04 | -2.62 | 0.009 | 0.89 (0.82 ~ 0.97) |
| INR | 0.09 | 0.04 | 2.23 | 0.026 | 1.09 (1.01 ~ 1.18) |  | 0.15 | 0.23 | 0.63 | 0.531 | 1.16 (0.73 ~ 1.82) |
| Gender, | 0.02 | 0.00 | 7.43 | <.001 | 1.02 (1.01 ~ 1.02) |  | 0.01 | 0.00 | 5.86 | <.001 | 1.01 (1.01 ~ 1.02) |

**Supplementary Table 3.Association Between HALP Score (as a Continuous Variable) and In-Hospital Mortality in the MIMIC-IV Cohort: Cox Proportional Hazards Analysis (Left-Side of the Threshold)**
