# Supplementary material for: Association between HALP score and in-hospital mortality in sepsis patients: a multicenter retrospective cohort study with external validation
Source: Front Public Health. 2026 Jan 12;13:1710118. doi: 10.3389/fpubh.2025.1710118 (PMC12832424; doi:10.3389/fpubh.2025.1710118)
Supplement: Supplementary file 7 [file Table_4.docx]

| Variables | Univariate | | | | |  | Multivariate | | | | |
| --- | --- | --- | --- | --- | --- | --- | --- | --- | --- | --- | --- |
|  | β | S.E | Z | P | HR (95%CI) |  | β | S.E | Z | P | HR (95%CI) |
| HALP | 0.00 | 0.00 | 1.41 | 0.158 | 1.00 (1.00 ~ 1.00) |  | 0.00 | 0.00 | 0.36 | 0.721 | 1.00 (1.00 ~ 1.00) |
| Gender |  |  |  |  |  |  |  |  |  |  |  |
| Male |  |  |  |  | 1.00 (Reference) |  |  |  |  |  | 1.00 (Reference) |
| Female | -0.02 | 0.09 | -0.16 | 0.873 | 0.99 (0.82 ~ 1.18) |  | -0.00 | 0.10 | -0.02 | 0.983 | 1.00 (0.83 ~ 1.20) |
| Hypertension |  |  |  |  |  |  |  |  |  |  |  |
| No |  |  |  |  | 1.00 (Reference) |  |  |  |  |  | 1.00 (Reference) |
| Yes | -0.18 | 0.10 | -1.84 | 0.065 | 0.83 (0.68 ~ 1.01) |  | -0.06 | 0.10 | -0.62 | 0.539 | 0.94 (0.77 ~ 1.15) |
| Diabetes mellitus |  |  |  |  |  |  |  |  |  |  |  |
| No |  |  |  |  | 1.00 (Reference) |  |  |  |  |  | 1.00 (Reference) |
| Yes | -0.02 | 0.10 | -0.15 | 0.878 | 0.98 (0.81 ~ 1.20) |  | -0.24 | 0.11 | -2.18 | 0.029 | 0.79 (0.64 ~ 0.98) |
| Age | 0.01 | 0.00 | 4.05 | <.001 | 1.01 (1.01 ~ 1.02) |  | 0.02 | 0.00 | 4.84 | <.001 | 1.02 (1.01 ~ 1.02) |
| WBC | 0.00 | 0.00 | 0.33 | 0.743 | 1.00 (1.00 ~ 1.01) |  | -0.00 | 0.00 | -0.70 | 0.485 | 1.00 (0.99 ~ 1.00) |
| Glu | 0.01 | 0.00 | 2.62 | 0.009 | 1.01 (1.01 ~ 1.01) |  | 0.00 | 0.00 | 0.53 | 0.595 | 1.00 (1.00 ~ 1.00) |
| Lactate | 0.14 | 0.01 | 11.54 | <.001 | 1.15 (1.12 ~ 1.18) |  | 0.09 | 0.01 | 5.78 | <.001 | 1.09 (1.06 ~ 1.12) |
| BUN | 0.01 | 0.00 | 5.28 | <.001 | 1.01 (1.01 ~ 1.01) |  | 0.00 | 0.00 | 0.99 | 0.322 | 1.00 (1.00 ~ 1.01) |
| Scr | 0.09 | 0.02 | 3.97 | <.001 | 1.09 (1.05 ~ 1.14) |  | -0.01 | 0.04 | -0.28 | 0.783 | 0.99 (0.92 ~ 1.07) |
| INR | 0.21 | 0.03 | 7.69 | <.001 | 1.23 (1.17 ~ 1.30) |  | 1.46 | 0.76 | 1.94 | 0.053 | 4.32 (0.98 ~ 19.02) |
| APSIII | 0.02 | 0.00 | 12.82 | <.001 | 1.02 (1.02 ~ 1.03) |  | 0.02 | 0.00 | 8.05 | <.001 | 1.02 (1.01 ~ 1.02) |

**Supplementary Table 4.Association Between HALP Score (as a Continuous Variable) and In-Hospital Mortality in the MIMIC-IV Cohort: Cox Proportional Hazards Analysis (Right-Side of the Threshold)**
