# Supplementary material for: Association between HALP score and in-hospital mortality in sepsis patients: a multicenter retrospective cohort study with external validation
Source: Front Public Health. 2026 Jan 12;13:1710118. doi: 10.3389/fpubh.2025.1710118 (PMC12832424; doi:10.3389/fpubh.2025.1710118)
Supplement: Supplementary file 8 [file Table_5.docx]

**Supplementary Table S5. Test of the proportional hazards assumption using Schoenfeld residuals in the primary multivariable Cox model**

| Variable | eICU Cohort (n=12,895) | | | MIMIC-IV Cohort (n=3,726) | | |
| --- | --- | --- | --- | --- | --- | --- |
|  | χ² | df | P-value | χ² | df | P-value |
| HALP | 2.14 | 1 | 0.144 | 0.88 | 1 | 0.349 |
| Age | 0.01 | 1 | 0.931 | 14.11 | 1 | 0.00017 |
| Gender | 0.51 | 1 | 0.475 | 0.48 | 1 | 0.489 |
| APS III / apachescore | 91.49 | 1 | <0.001 | 59.95 | 1 | <0.001 |
| GCS | 25.03 | 1 | <0.001 | 0.28 | 1 | 0.599 |
| Mechanical ventilation | 16.32 | 1 | <0.001 | 1.95 | 1 | 0.163 |
| Creatinine | 4.28 | 1 | 0.039 | 8.59 | 1 | 0.003 |
| Lactate | 42.71 | 1 | <0.001 | 48.17 | 1 | <0.001 |
| **Global** | **119.27** | **8** | **<0.001** | **96.16** | **8** | **<0.001** |

Test of the proportional hazards assumption using Schoenfeld residuals in the primary multivariable Cox model (adjusted for HALP, age, gender, APS III/apachescore, GCS, mechanical ventilation, creatinine, and lactate). The HALP score itself satisfied the assumption in both cohorts (both P>0.10).
