# Supplementary material for: Association between HALP score and in-hospital mortality in sepsis patients: a multicenter retrospective cohort study with external validation
Source: Front Public Health. 2026 Jan 12;13:1710118. doi: 10.3389/fpubh.2025.1710118 (PMC12832424; doi:10.3389/fpubh.2025.1710118)
Supplement: Supplementary file 9 [file Table_6.docx]

| **Supplementary Table S6. Sensitivity analyses for the association between high HALP and in-hospital mortality after addressing non-proportionality of lactate** |  |  |  |  |  |
| --- | --- | --- | --- | --- | --- |

| Model | Cohort | High HALP vs Low HALP | HR | 95% CI | P-value |
| --- | --- | --- | --- | --- | --- |
| Primary model (adjusted for lactate) | eICU | High HALP (reference: Low HALP) | 0.91 | 0.84–0.99 | 0.048 |
|  | MIMIC-IV | High HALP (reference: Low HALP) | 0.88 | 0.77–1.00 | 0.067 |
| **Lactate tertile-stratified model** (recommended) | eICU | High HALP | **0.94** | 0.86–1.02 | **0.135** → borderline |
|  | MIMIC-IV | High HALP | **0.87** | 0.75–0.99 | **0.044** |
| Exclusion of lactate | eICU | High HALP | 0.91 | 0.83–0.99 | 0.041 |
|  | MIMIC-IV | High HALP | 0.87 | 0.76–1.00 | 0.058 |

Sensitivity analyses for the association between high HALP and in-hospital mortality after addressing non-proportionality of lactate. All models were adjusted for age, gender, APS III/apachescore, GCS, mechanical ventilation, and creatinine.
