# Supplementary material for: Association between HALP score and in-hospital mortality in sepsis patients: a multicenter retrospective cohort study with external validation
Source: Front Public Health. 2026 Jan 12;13:1710118. doi: 10.3389/fpubh.2025.1710118 (PMC12832424; doi:10.3389/fpubh.2025.1710118)
Supplement: Supplementary file 10 [file Table_7.docx]

**Supplementary Table S7. Competing-risk analysis using Fine-Gray subdistribution hazard model (live discharge treated as competing event)**

| Cohort | High HALP vs Low HALP | Subdistribution HR | 95% CI | P-value |
| --- | --- | --- | --- | --- |
| eICU (n=12,895) | High HALP (reference: Low HALP) | 0.915 | 0.835–1.002 | 0.057 |
| MIMIC-IV (n=3,726) | High HALP (reference: Low HALP) | 0.878 | 0.758–1.017 | 0.082 |

All models adjusted for age, gender, APS III/apachescore, GCS, mechanical ventilation, creatinine, and lactate.HR < 1 indicates lower cumulative incidence of in-hospital death in the presence of the competing risk of live discharge.
